# Trauma-Directed Interaction (TDI): An Adaptation to Parent-Child Interaction Therapy for Families with a History of Trauma

**DOI:** 10.3390/ijerph19106089

**Published:** 2022-05-17

**Authors:** Robin H. Gurwitch, Christina M. Warner-Metzger

**Affiliations:** 1 Department of Psychiatry and Behavioral Sciences, Duke University Medical Center, Durham, NC 27701, USA; robin.gurwitch@duke.edu; 2 Evidence-Based Practices and International Consulting (EPIC), LLC, Chicago, IL 60605, USA

**Keywords:** parent-child interaction therapy, trauma, families

## Abstract

Parent-Child Interaction Therapy (PCIT) is one of the strongest evidence-based treatments available for young children and their families. Research has supported the use of PCIT for children with a history of trauma; however, the treatment does not directly address trauma in the child. PCIT is a dyadic treatment; yet, the impact of the carer’s trauma on the carer-child relationship is not assessed or incorporated into treatment. For these reasons, therapists, families, agencies, and funders tend to view PCIT as a trauma treatment with skepticism. PCIT therapists who currently address trauma within the intervention do so without a standardized approach. Trauma-Directed Interaction (TDI) is an adaptation developed to directly address these concerns. TDI maintains the key elements and theoretical underpinnings of PCIT while adding sessions to cover psychoeducation about trauma, carer response to a child’s trauma reactions (SAFE skills), and coping skills to aid both the child and the carer to manage trauma activators (COPE skills). The TDI module creates a consistent strategy for PCIT therapists to address trauma, thus allowing research and replication which will advance the dual fields of PCIT and family trauma. The theoretical conceptualization of TDI is presented along with next steps in its evaluation.

## 1. Parent-Child Interaction Therapy (PCIT) and Trauma

Parent-Child Interaction Therapy (PCIT) is considered one of the strongest evidence-based treatments for young children (2–7 years) and their carers [1], who are defined as anyone (including young adults and adults) who serves the child in a caregiving role, such as biological, kinship, adoptive, and foster care [2]. Developed in the mid-1970s by Sheila Eyberg [3], PCIT’s theoretical underpinnings are based upon Baumrind’s parenting models [4,5], play therapy [6], social learning theory [7], attachment theory [8], and Patterson’s coercive cycle of parenting [9]. Key components of PCIT include coding the carer-child interaction at each session to assess progress [10], tracking weekly child behavioral symptoms [11], and in vivo (live) coaching of the carer by the therapist. This unique feature of PCIT is a strength, as live skills coaching has been found to improve treatment outcomes [12]. PCIT contains two phases in the treatment as initially advocated by Hanf [13]: Child-Directed Interaction (CDI) and Parent-Directed Interaction (PDI). The first phase of PCIT is focused on creating a strong and secure attachment between the carer and the child through the carer’s use of skills designed to improve the positive relationship and warmth, laying the foundation for the second phase of PCIT. In PCIT, these skills are known as PRIDE skills (Labeled **P**raise, **R**eflection, **I**mitation, Behavior **D**escription, and **E**njoyment). In PDI, consistency, predictability, and follow-through are stressed as carers are taught and coached in the implementation of a positive discipline program that can be used across settings. In both phases, carers are coached to reach skills criteria, which is one way to identify the phase goals have been achieved. As included in the PCIT protocol session integrity checklists [3], the progress is shared and discussed with carers each session, thus reinforcing the treatment as a collaborative and transparent endeavor between the carer and therapist.

With over 300 scientific articles and book chapters on PCIT [14], studies show consistently positive outcomes. PCIT reduces both externalizing (disruptive) behaviors and internalizing problems [15,16,17]. Reductions in behavior problems have been found when PCIT is used with young children with fetal alcohol spectrum disorders [18] and prenatal substance exposure [19], children with autism spectrum disorders [20,21], and children with developmental delays [22]. The positive effects of PCIT have been shown to generalize to untreated siblings [23] and to school settings [24]. In-home delivery [25], as well as delivery through internet sessions [26], have been effective. The latter has been especially timely with the onset of COVID-19 propelling mental health treatment via the internet as the primary mode of delivery [27]. PCIT’s positive results have also been found to reduce caregiving stress [28] and increase carer positive affect when interacting with their children [29].

While trauma adversely affects relationships [30], PCIT results in more positive carer-child relationships [28]. However, when PCIT was developed in the early 1970s, the *Diagnostic and Statistical Manual* did not have a diagnosis of PTSD, which first appeared in 1980 [31]. A young child’s exposure to trauma is often associated with negative behaviors and attachment issues [32]; therefore, it is highly likely that PCIT therapists were often treating cases of children with a history of trauma. This is highly probable since the peak ages for child maltreatment are in the preschool and early school-age years, the targeted age-range for PCIT [33]. As trauma is discussed in this article, the definition of trauma developed by the Substance Abuse and Mental Health Services Administration (SAMHSA) [34,35] will apply: “individual trauma results from an event, series of events, or set of circumstances that is experienced by an individual as physically or emotionally harmful or life threatening and that has lasting adverse effects on the individual’s functioning and mental, physical, social, emotional, or spiritual well-being”. Indeed, more than half of children in the United States (approximately 46 million) are exposed to violence, crime, and abuse each year [36]. Yet, robust scientific findings reveal at least one positive adult relationship in a child’s life is a key factor in helping children build resilience against adversity [37,38,39,40].

The effectiveness of PCIT with children who have experienced trauma has been well-documented in the scientific literature [41,42,43,44,45]. In a seminal randomized controlled trial, Chaffin and colleagues [42] found for carers who were abusive, PCIT reduced child maltreatment recidivism at 2.5 years to less than 20% relative to carers in standard treatment who experienced rates of almost 50% recidivism. Furthermore, in a separate study examining a community sample, Pearl and colleagues [46] found a reduction of trauma symptoms for children receiving PCIT. All subscales on the Trauma Symptom Checklist for Young Children (TSCYC) [47] fell to the subclinical range by posttreatment, with anger, anxiety, arousal, intrusion, dissociation, and overall post-traumatic stress symptoms showing medium to large effect sizes. Because of the science supporting the multi-faceted impact of PCIT with the trauma population, several trauma-informed entities and funders endorsed PCIT for treating children with trauma. The Kaufman Best Practices Report [48] identified PCIT as one of the three best treatments for children with a history of maltreatment. Moreover, the National Traumatic Stress Network [49] lists PCIT among its recommended treatments for child maltreatment and the California Evidence-Based Clearinghouse for Child Welfare [1] gives PCIT its highest ranking for scientific support. With such robust evidence for effectiveness, PCIT was the first treatment the Family First Prevention Services Act (FFPSA) [50] endorsed specifically for young children. The FFPSA describes PCIT as favorably impacting child behavioral and emotional functioning, positive carer practices, and carer mental health [50].

## 2. The Necessity of Trauma-Directed Interaction (TDI)

Given that PCIT works for children with a history of trauma, why would an adaptation specific to trauma be necessary? While PCIT addresses the *family relationships* often adversely impacted by trauma history, the treatment does not specifically address the *child’s trauma*. Inherent in the PCIT protocol, there is no psychoeducation related to trauma to help carers better understand the impact of such experiences on young children or on themselves. Yet, science has well established exposure to trauma in young children is associated with both externalizing and internalizing behavior problems, including oppositional and defiant behaviors, anxiety, and aggression [51,52]. Therefore, disruptive behaviors secondary to underlying trauma may influence the referral of a child to PCIT. 

The “I” in PCIT stands for interaction. While PCIT is a strong dyadic model with an emphasis on the relational interaction, the bi-directionality of trauma exposure is left relatively absent from treatment. Consequently, *carer traumatic experiences* and the *intergenerational transmission of trauma* are also not addressed in PCIT but are ever-growing concerns for the global community. The Center for Disease Control and Prevention [53] estimates one in four women will experience domestic violence each year resulting in roughly 5.3 million incidents. With the social isolation of COVID-19, the rates of domestic violence have increased 25% to 33% globally, with similar increases reported in cities in the United States [54]. As adults, child witnesses of domestic violence may replicate the abusive pattern in their own interpersonal relationships [55], resulting in intergenerational trauma. Domestic violence has also been found to negatively affect carer well-being with increased levels of depression, anxiety, somatization, and social isolation [56,57]. Likewise, the carer-child relationship may be adversely affected by domestic violence [58,59]. Mothers who have experienced domestic violence may be less emotionally available to their children due to their life stress and heightened emotions [60] and may not feel confident in their caregiving abilities [61]. Their caregiving style has been characterized as inconsistent, unemotional, ineffective, reactive, and punitive [60,62,63,64]. Some women who experience interpersonal violence may also show less warmth towards their children [65]. 

Domestic violence is but one example of carer trauma. Carers may have their own history of child maltreatment, substance abuse issues, and/or exposure to violence, and when combined, this may create a cumulative trauma history that impacts caregiving functioning [66]. The Adverse Childhood Experiences (ACEs) study [67] assessed adult exposure to 10 different traumatic events during childhood, including all forms of maltreatment, substance misuse, carer mental health problems, domestic violence, and relative incarceration. Findings from this study indicated ACEs are associated with health (e.g., diabetes, cancer, smoking, substance abuse), mental health (e.g., depression, suicide), and lifestyle problems (e.g., problems at work/school, homelessness, unemployment, trouble with the law) in childhood and into adulthood. A recent survey found over 60% of adults across 23 states reported at least one ACE, while 25% reported 3 or more [68]. Four or more ACEs seem to place a child at high vulnerability for the adverse outcomes as adults [67]. However, the original ACEs study was limited to a majority White, female, 40 and older, college educated, and insured population. Recently, the types of ACEs have been expanded to include exposure to adverse community environments (e.g., poverty, discrimination, lack of opportunities, poor housing, and violence) [69]. People of color report more ACEs than White populations [70,71,72]. This is further compounded as stress due to discrimination is also associated with more overall adverse health outcomes [73]. Exposure to trauma is associated with more harsh approaches to caregiving and a higher likelihood of child neglect [74,75]. Carers who are diagnosed with Post-Traumatic Stress Disorder (PTSD) report lower caregiving satisfaction [76] and children with carers having PTSD are more likely to develop emotional and mental health problems [77]. In sum, a carer’s trauma has a significant and profound impact on the effectiveness of their caregiving and their carer-child relationship [66]. 

In 2022, any discussion of trauma would be incomplete without examining the impact of the pandemic on the mental health of children and carers. Indeed, COVID-19 has been conceptualized in the literature as a potentially traumatic event [78,79]. There has been an increase in adult reports of anxiety, depression, substance use [80], and Post-Traumatic Stress Disorder (PTSD) [81]. This increase is more pronounced in carers [82], including findings of problematic carer-child psychological well-being in families with young children in the PCIT age range of 2–7 years [83]. Research indicates a similar pattern in children, with increases in adverse mental health outcomes mirroring those in their carers [84,85]. In particular, childhood reactions to the pandemic show increases in problematic behaviors [86] and in visits to the emergency room because of behavioral health issues for children, including for children as young as 5 years [87]. The escalation in mental health concerns in youth has led to a 2021 U.S. Surgeon General’s Advisory [88] to address this crisis. 

Furthermore, due to COVID-19, an estimated 140,000 children in the United States are likely to experience the death of a carer, which qualifies as a traumatic event for a young child [89]. Losing a carer may adversely affect a young child’s view of the world as a secure and safe place, as well as disrupt a child’s ability to form secure attachments to carers, putting them at risk for mental health problems [90,91]. Complicating the grief process, the remaining carer also experiences their loss along with the child’s loss, again underscoring the bi-directionality of relationships. In addition to death, children and families have experienced an increase in housing, food, and financial insecurities, as well as disruptions in childcare and education, resulting in increased stress in all family members [88]. All of these factors have been more prevalent in families of color who are also more likely to have experienced trauma [92]. With PCIT being an identified evidence-based treatment for families with young children who have mental health concerns [50] and as more families are seeking mental health services [93], explicitly incorporating a trauma component that underscores the bi-directionality of relationships seems especially important at this time. 

Previous research clearly upholds PCIT as an appropriate treatment for young children (2–7) with a history of trauma [45] and for carers who have experienced significant ACEs [94]. Publication records and PCIT International Biennial Convention and World Congress agendas document almost two decades of topical articles, chapters, presentations, and workshops expanding the therapy to childhood trauma issues. In 2018, FFPSA [50] endorsed PCIT as a well-supported trauma-informed mental health treatment for children. However, perceptions persist within therapists, families, referral sources, and community and state entities that PCIT is not a specified “trauma treatment”, therefore, it cannot be effective [95]. While other treatments were developed specifically for trauma (e.g., child-parent psychotherapy, trauma-focused cognitive behavior therapy) [91,96], PCIT was initially developed for children with disruptive behaviors [3]. Accordingly, many therapists, agencies, and funders are more likely to seek trauma-specific treatments [95]. These obstacles hinder research and dissemination of PCIT as an effective trauma treatment and present unique challenges when procuring buy-in from stakeholders and funders who want an “overt” commitment to addressing trauma within a therapy model. Thus, opportunities are missed for effective trauma treatment during a critical developmental period.

In a recent study to ascertain the perceptions of PCIT International Certified Therapists and their PCIT practice with the trauma population, almost 80% of the sample who completed an online survey indicated a supplemental trauma-based module seems needed to various degrees, ranging from necessary to very necessary [97]. Respondents further identified the trauma module should include standardized psychoeducation for carers, trauma assessment for carers and children involved in treatment, and content related to safety and security. In terms of timing and integration, most (39.2% of) PCIT therapists suggested including the trauma module before starting PCIT, followed by integrating the trauma module throughout PCIT (33.8%), and inserting the trauma module between the CDI and PDI phases of PCIT (21.5%).

Although many therapists incorporate trauma-specific content into their PCIT practice, to date, no consistent or standardized approach has been developed. In effect, rather than using the masses to create “an n (sample size) of one million”, therapists’ siloed attempts create “a million n’s of one”, or several individual case studies with varied approaches. While this may benefit individual families in an isolated manner, it creates numerous barriers to replication; to communication across therapists, trainers, community agencies, and referral sources; and, finally, to congruence for treatment integrity.

## 3. TDI: An Adaptation and Its Goals

The Centers for Disease Control and Prevention defines “fidelity” as the “faithfulness with which a curriculum or program is implemented, without compromising its core components” [98]. In 2005, the PCIT developer (Eyberg) [99] outlined several core components of PCIT, including (a) carers and children together attend and engage in sessions, (b) therapists code carer-child interactions, (c) therapists use live coaching to teach positive caregiving via specified child-directed and carer-directed skills, and (d) carers learn broad classes of antecedents and response behaviors. Moreover, three levels of strategic changes to treatment protocol, each with varying degrees of impact on treatment fidelity, were identified: (1) tailoring, (2) adapting, and (3) modifying [99]. Tailoring is defined as “changes made in the focus or delivery style of essential elements in an established treatment based on the unique features of the individual case” (p. 199). It occurs when “differences in family attitudes, history, beliefs, or behaviors relevant to the therapeutic process require changes in treatment” (p. 199). As an example of tailoring to individual family differences, recent strides in internet-delivered PCIT [26,100] changed the delivery of PCIT from traditional on-site therapy to telehealth sessions while maintaining the core components of PCIT. This tailoring research proved essential in the midst of the COVID-19 pandemic when telehealth was critical to helping families continue or begin mental health treatment [101] and allowed a pivot across PCIT International to continue to provide an effective intervention [27]. Likewise, MY PCIT [102] personalizes the implementation of PCIT by standardizing assessment of culturally-influenced factors. This in turn allows the clinician to tailor the use of personalization tools, such as informational handouts, to match each family’s Parent Explanatory Model (PEM) to PCIT and increase treatment engagement. In light of the social justice movement, MY PCIT is a tailoring of treatment that addresses issues important to families. 

Adaptation refers to “changes in the structure/content of an established treatment, typically made when aspects of the standard treatment are not feasible or sufficient in the new population” [99] (p. 200). Adaptations occur rarely, under expert consultation, and within researched protocols. Adaptations, including Bravery-Directed Interaction (BDI) [103] and Guiando a Niños Activos (Guiding Active Children, or GANA) [104], often add components to PCIT to more aptly address the needs of specialized populations while maintaining core elements (i.e., coding, coaching, and skills criteria) of PCIT. 

The final level of treatment change is the most extensive, extremely rare, and requires substantial research to support it. Modifying is known as “universal changes in established treatments, made by the treatment developer, typically resulting from treatment component research” [99] (p. 200). A recent example of PCIT modification is PCIT Toddlers [105,106] in which PCIT is modified in a downward extension to the developmental level of children ages 12 to 24 months. PCIT Toddlers adds emotion regulation skills and substantially changes PCIT procedures for managing aggressive and destructive behaviors, as well as implementing carer-directed skills that prioritize scaffolded learning principles over discipline with consequences for this youngest age range. 

As an adaptation of PCIT, the Trauma-Directed Interaction (TDI) module was developed into a manualized intervention with several goals in mind. Having a strong positive relationship is considered a protective factor for children who have experienced trauma [107] and the carer-child relationship has been posited as the most salient factor in reducing children’s symptoms following trauma [108]. Already a key element in PCIT, strengthening the carer-child relationship is also stressed in TDI as an essential goal. Through the TDI teach and coaching sessions, carers understand the impact of trauma on child behaviors and on relationships. Relatedly, carers learn to identify and differentiate between child upset and child trauma activators. Another goal for TDI is for carers to develop effective and consistent responses to address trauma activators. When children are upset for a variety of reasons, having skills to cope with the upset is important. Furthermore, as carer trauma also impacts caregiving and relationships, this module helps carers recognize their own trauma activators and the contribution of trauma (the child’s and/or their own) to caregiving stress and caregiving abilities. The final goal of this module is to provide coping skills for use by the child and carers. These goals are accomplished in a manner similar to the other two phases (CDI and PDI) of PCIT: a TDI teach session, homework to practice new skills, and in vivo coaching sessions. However, unlike skills criteria for the other phases, TDI has a set number of sessions (four), with continued incorporation of skills throughout the remainder of PCIT. See Table 1 for a comparison of standard PCIT components and the TDI adaptation components.

## 4. TDI Components

### 4.1. TDI Teach

As with CDI and PDI teach sessions, the TDI teach session is a carer-only session. This allows the carer’s focus to be solely on the information being presented and allows for all questions and concerns to be addressed, without interruption or worry about appropriateness of discussion for the young child to overhear. Using concepts similar to MyPCIT [102], the TDI adaptation recommends a personalized discussion of carer perceptions of the PCIT and TDI skills as they are presented during the teach session. Thus, TDI flexibly adjusts to cultural aspects of the skills during coaching and throughout treatment to promote healthier family relationships. This is important as cross-cultural dimensions related to caregiving [109] may impact outcomes in treatment. The TDI teach covers four main areas specific to trauma: (1) trauma psychoeducation, (2) differentiation between child upset and trauma responses, (3) carer responses to trauma activators, and (4) child responses to trauma activators.

#### 4.1.1. Trauma Psychoeducation

The trauma psychoeducation component of TDI may elicit the carer’s trauma activators of their personal trauma and their child’s trauma, including historical trauma, marginalization, and oppression. Therefore, therapists are encouraged to be mindful of and sensitive to the family’s legacy of trauma as they proceed with the psychoeducation. With the TDI teach, the child’s trauma is directly named and addressed with examples of its significance cited throughout the session. Naming the trauma is a key element in trauma treatments for both children and adults [91,96]. As part of the TDI teach, carers learn about different types of trauma (e.g., single, chronic, complex, and historical). They also are taught about how stressors in the home, community, and/or in larger systems (e.g., substance abuse, frequent placement disruptions, racism) may influence a child’s sense of safety, security, and attachment. The family’s unique trauma experiences are interwoven into this TDI teach session.

Based on scientific research, the TDI teach covers the developmental nature of childhood trauma. One topic for this section includes how the brain and child development are affected by trauma, discussing how the adverse consequences of trauma may include problems with emotion regulation and learning/remembering information [110]. A second area of discussion during the session is how trauma experiences in young children are often expressed through their behaviors [45], using examples noted at intake and throughout the CDI phase of treatment to personalize the TDI teach. Examples include irritability, hyperactivity, impulsivity, defiance, and noncompliance; these are often the behavioral concerns that initially brought the family into treatment. A final topic in this portion of the TDI teach involves trauma’s adverse impact on relationships: undermining a child’s sense of trust, security, and safety, as well as their ability to identify and respond to emotional cues [91]. The gains made in CDI are reviewed and carers are congratulated on how their use of CDI skills helps to address concerns related to trauma and to move the trajectory for the child and the carer-child relationship in a positive direction. The TDI phase is the next important step in this process.

In standard application of PCIT, the carer’s trauma history and/or the impact of the child’s trauma on the carer is neither assessed nor addressed, including historical trauma and racism. In TDI, all are discussed as carer trauma and how it may adversely affect the relationship [94,111]. Experiences with interpersonal violence, own childhood history of trauma, and community violence are all too common in families seen for mental health treatment [112,113]. The TDI teach engages carers in reflections about their own childhood, including ethnic and cultural practices related to behaviors, and how their child’s reactions to trauma activators may remind them of their own trauma and elicit similar reactions. To further help carers identify trauma reactions in their child or themselves, the concept of trauma activators is discussed. These may be sights, smells, people, places, activities, and/or behaviors that remind a person of the trauma and increase emotional upset, making it sometimes difficult to calm. A worksheet is completed with the carer, identifying activators for both the child and the carers. 

#### 4.1.2. Differentiate Upset from Trauma Activation

Not all disruptive behaviors or emotional upset are related to trauma. For children with a trauma history, it is understandable for carers to often assume negative behaviors, including temper tantrums, are trauma related. To help carers distinguish the difference, a discussion of upset vs. trauma activation is included in the TDI teach. The connection between antecedents and behaviors is shared [96]. For example, is the child responding to limits, rules, and not having a want granted (e.g., “I want the toy you are playing with right now!”) or is the child responding to a possible trauma activator (e.g., a child who has experienced gun violence reacts to the loud sound of a car backfire)? By helping carers differentiate possible precursors and symptoms of upset versus trauma activation during the TDI teach, this knowledge may then be incorporated into the TDI coach sessions and beyond. TDI allows carers to more effectively identify and respond to the behaviors. 

In TDI, carers are taught to recognize children’s reactions to distress. Furthermore, carers are taught and coached to react to the distress in a consistent and predictable manner. In CDI, carers establish the fundamental elements of consistent therapeutic play routines, a predictable set of interaction skills, and initial boundaries on acceptable behavior. In TDI, these essential practices are expanded to not only help attenuate the child’s distress, but to also reduce carer distress with planned responses. Carers are taught the exact words to say when trauma activators arise to allow consistent responses across various settings. This consistency and predictability are two other elements that help reduce the impact of trauma and build resilience in children [114]. These habits created in CDI are strengthened in TDI and are further reinforced in PDI.

#### 4.1.3. Subjective Units of Distress (SUDS)

To monitor progress in PCIT with TDI, along with the data collected to track progress within PCIT (i.e., weekly Eyberg Child Behavior Inventory [ECBI] [11], weekly homework, and session-by-session Dyadic-Parent Child Interaction Coding System [DPICS] [10]), TDI incorporates the use of the Subjective Units of Distress Scale (SUDS) [115] for a brief assessment of carer distress within and across sessions. SUDS ratings are often used within the therapeutic literature [96,116] to increase self-awareness of an individual’s current state of distress on a Likert scale typically ranking from 0 (representing peace and complete calm or totally relaxed) to 10 (representing unbearably upset to the point of total dysfunction or highest distress ever felt). Similar to using a “fear thermometer” for child self-report in BDI [103], SUDS is first introduced as a carer self-assessment tool during the TDI teach and used several times during the session as concepts related to childhood and carer trauma are discussed. SUDS ratings are also gathered throughout the TDI coaching sessions and integrated into the PDI phase of treatment. Obtaining carer SUDS ratings helps the carer assess their own distress and monitor how their coping strategies to lower distress are working. Furthermore, SUDS ratings also remove the guesswork of determining the carer’s emotional state, providing rich data to guide therapist coaching strategies based on the carer’s *experienced* levels of distress, perhaps preventing the therapist from emotionally overcompensating for *perceived* distress. For example, a carer who looks away from their screaming child and places their hand near their forehead, covering their face from the child’s view may be *perceived* by the therapist as moderately “upset”, while the carer’s SUDS rating of a 2 indicates the carer is *experiencing* minimal distress and perhaps hiding their face in good humor at the child’s antics in trying to have demands met.

#### 4.1.4. Carer Response: SAFE Skills

In TDI, carer responses to child distress are identified as the SAFE skills: **S**tate the feeling, **A**ddress safety, **F**ind a COPE skill, and **E**ngage in PRIDE skills.

State the Feeling. Often, young children may not have a sufficient vocabulary to name their feelings and negative behaviors result when they are misunderstood [117,118]. With the S in SAFE, carers scaffold and help their children to state or name the feeling, thus building children’s emotion vocabulary. Carers are taught to validate the emotion rather than “fix it.” Carers learn exact words to use to (a) label the emotion, (b) validate the emotion, and (c) connect the name of the emotion to the feelings associated with it. With this SAFE skill, carers are also encouraged to identify their own emotional states and to begin recognizing a pattern between the child’s emotion and their own.

Address Safety. When children experience trauma, their sense of safety is often undermined [91,110,113]. In TDI, it is important to reassert the idea of safety with both words and actions. This increases the child’s identification of their carers as trusted adults, which, in turn, improves coping and resilience. Again, carers are given words to say each time a feeling associated with traumatic play or traumatic activation is noted. This SAFE skill stresses the child is now safe and the carer is available to the child. As with CDI and PDI, it is expected with the consistency of the words, children will internalize these scripts and begin to repeat them; this may serve as a calming “mantra” for the child. As done during the CDI and PDI teach sessions, role-plays using the scripts are included during the TDI teach session so the carer becomes accustomed with application to different scenarios (e.g., animals fighting, toy figures yelling at each other, cars crashing, an animal being separated from others, or towers being knocked over or pieces scattered). Coaching reinforces how to consistently address safety throughout remaining sessions.

Find a COPE Skill. As will be discussed below, carers and children are taught a variety of coping skills. When a child is distressed, the carer is instructed to remind the child to use one of the learned skills. The carer then practices it with their child and offers labeled praise (a CDI skill) for the effort in employing the positive coping strategy.

Engage in PRIDE Skills. After implementing the first three SAFE skills, the carer is coached to return to the familiarity of the PRIDE skills learned in CDI. TDI does not process the child’s trauma in depth. Once the SAFE skills are used, discussion of the trauma and the reaction are concluded, and the carer and child return to the consistency and normalcy of CDI (i.e., engage in PRIDE). This ritual is expected to be calming to both the child and the carer and to reinforce the strength of their relationship. Carers are encouraged to find opportunities outside of PCIT special time to praise the child for calm, positive actions and behaviors, and for sharing possibly distressing information with the carer. Again, during the TDI teach, carers and the therapist role-play this engagement in PRIDE skills, with further coaching in subsequent sessions.

The carers’ lived experiences with trauma are also recognized. As each of the SAFE skills are introduced, there is a brief discussion of how it may apply to the carer’s own reactions to trauma activators. Their comfort and abilities to use the SAFE skills are continually assessed during the TDI teach and the coaching sessions. In addition, as SAFE skills are implemented, SUDS ratings assist the therapist in monitoring the carer’s regulation, with the expectation the SUDS levels will decrease within the session and over time.

#### 4.1.5. Child Response: COPE Skills

Many evidence-based treatments for mental health concerns related to trauma include the introduction and support of coping skills to enhance healing and build resilience in the face of traumatic activators [119,120,121]. TDI includes the introduction of such skills for both the child and the carer. Coping skills temper negative reactions elicited by trauma reminders, helping with both negative behaviors and distressing emotions [96]. TDI coping skills are taught to the carer. They are coached to use these skills with their child and to also use these skills themselves when experiencing negative reactions. In CDI, it is common for young children to model skills and language used and/or reinforced by carers. As carers use TDI skills, it is expected similar modeling will occur. Overall, the COPE skills are expected to reduce negative reactions due to trauma reminders and trauma activators, as well as at other times of distress for both the child and the carer.

As the SAFE skills represent how carers are taught to react to their child’s trauma reactions, COPE is the acronym in TDI to represent new coping skills for the family to use. COPE stands for **C**olor breathing, **O**pen to feelings, **P**ositive action, and **E**xpress yourself. These skills are briefly discussed in the TDI teach, with the rationale for each skill provided. 

Color Breathing. Relaxation skills are included in numerous adult and child treatment programs for trauma [96,122]. Relaxation is a way for the body to calm when it is in a state of stress, hypervigilance, and/or undue arousal (e.g., worry, anxiety, anger) resulting from a traumatic event or undue stress, which may or may not be trauma related. Consistent with other relaxation breathing techniques [96], color breathing works by slowing breathing and alternating focus to relax different parts of the body, creating a calming effect. Color breathing is a relaxation technique first developed for Healing After Trauma Skills (HATS) created in the aftermath of the bombing of the Alfred P. Murrah Federal Building in Oklahoma City in 1995 [123] as part of an intervention designed to reduce negative reactions in young children to this event. Since that time, color breathing has been used in newer versions of HATS [117] by the American Red Cross [124] and in activities of the National Child Traumatic Stress Network after traumatic events [125]. The application of color breathing within TDI provides young children with a developmentally appropriate mechanism to calm and gives PCIT therapists a standardized approach to help families presenting with trauma relax when stressed. 

Open to Feelings. The O in COPE seeks to expand emotion identification in young children whose feelings vocabulary is relatively limited [118]. Increasing this vocabulary improves communication with carers, as well as provides the child with words to use rather than simply reacting to distress with negative behaviors. Labeling feelings is used in trauma treatments [91,96,117] as one way to help with healing and resilience. Using a feeling faces handout [117], carers are taught to combine a feelings face with an example of when they experienced the feeling. For example, for the worried face, the carer may say, “I feel worried when people are fighting”. Carers are taught and then coached to combine both distressing feelings with happy ones (e.g., “I feel good when people get along”). Faces are divided by developmental age to assure understanding by the child. 

Positive Action. When experiencing distress due to trauma or upset, engaging in a positive action provides an alternative to negative feelings, thoughts, and/or behaviors [96]. Intrusive thoughts are common in adults who experience trauma and trauma activators are reported in both adults and children [27,116]. Creating a menu of positive actions redirects both thoughts and unhelpful actions. TDI posits that creating a predetermined menu of positive actions increases the likelihood those positive actions will be used by both carers and children. Collaboratively with the therapist, carers create a menu of possibilities for themselves and their child. PCIT’s special time and PRIDE skills are automatically included in the list, as are the C and O of COPE. When introducing the concept of positive action to the child, the menu may be further expanded. Carers are coached to engage in positive actions when distressing occasions arise. As children spontaneously use one of the actions, carers are encouraged to use their PRIDE skills to reinforce this resilience-building behavior. 

Express Yourself. Throughout the TDI teach session, the importance of a safe and secure relationship with the carer is emphasized; such a relationship will help to generalize to other relationships and to the world around the child [91]. The SAFE skills give carers a mechanism to respond to trauma-related feelings and reactions while validating the child’s emotions. It is with these goals and action steps in mind that the E in COPE, express yourself, was created. For children who have experienced trauma, it is essential they believe there will be someone to listen to them and to ensure their safety, within a loving and caring environment. Carers are taught to use the COPE skills should they incur distress hearing about their child’s trauma or responding to their child’s sharing about or reactions to the trauma. They are taught to use PRIDE skills when children share information, again reinforcing a sense of safety. Together, COPE helps to build resilience in the child as well as the carer. See Table 2 for a summary of TDI teach components.

### 4.2. TDI Session Progression

#### 4.2.1. From TDI Teach to TDI Coaching

During the TDI teach, the SAFE skills are discussed in-depth and all COPE skills are introduced at a cursory level to set the stage for the overall structure and purpose of TDI. However, the homework following the TDI teach session focuses on the carer first increasing self-awareness of their own feelings related to their child’s behavior throughout the week while continuing special time practice consistent with CDI. Subsequent to the TDI teach, the family completes a time-limited series of three TDI coach sessions. Each session introduces at least one new COPE concept, providing standardized language and opportunities for the family to practice the skills with the therapist. TDI homework builds in a cumulative fashion across the TDI coach sessions ending with all COPE skills introduced, modeled, and practiced by the third TDI coach session. The SAFE skills are used across the TDI coach sessions and integrated into homework. See Table 3 for a summary of the TDI session progression.

Limiting the TDI phase to a single teach session and three subsequent coach sessions retains the spirit of PCIT as a short-term therapy. Essentially, TDI inserts a four-week transition from the CDI phase to the PDI phase of treatment to allow for TDI introduction and initial implementation. A gradual roll-out for TDI facilitates incremental uptake of skills with supportive coaching to reinforce carer skills used to address trauma symptoms. Holding a series of TDI coach sessions not only increases opportunities to recognize child upset versus trauma activators, but also maximizes time for the carer to learn and to practice the TDI skills and for the child to internalize COPE concepts. The time-limited TDI phase also provides additional opportunities for continued CDI skills and relationship growth prior to moving into the positive discipline phase, PDI. 

A check-in occurs at the beginning of all PCIT sessions, including TDI. In a parallel process consistent with PCIT, carers and children are reinforced by the therapist with labeled praise when they share their use of the SAFE skills and COPE skills. As each COPE skill is introduced at the beginning of the session, there is in vivo practice followed by coaching integrated into the typical CDI components of the session (see Table 4). The COPE skills are taught in a cumulative manner across sessions and incorporated into subsequent PCIT special time practice in sessions and home practice. CDI special time home practice is extended by 5 min to allow for TDI skills practice. As with both phases of standard PCIT, TDI homework review tracks practice of the COPE skills. Most importantly, as the TDI skills are expected to further strengthen the carer-child relationship while incorporating a trauma lens, continued assessment of overall progress is tracked. Both COPE and SAFE skills are used, as opportunities allow, and coached throughout the remainder of PCIT. 

Beginning in the TDI Coach 1 session, color breathing [117] is taught in detail and practiced. With color breathing, carers are not only instructed to practice the relaxation skill when starting daily special time, but also once daily outside special time to accelerate skill acquisition. Open to feelings is introduced to the child at the beginning of TDI Coach 2 and incorporated throughout the remainder of PCIT. The homework assignment combines color breathing to start special time and open to feelings to end special time, as well as other generalization practice. Both positive action and express yourself are introduced at the start of TDI Coach 3, which is the final session of the TDI phase. After collaboratively generating a menu of options for calming activities with the family, they are assigned homework to continue with color breathing and open to feelings practice surrounding special time, as well as integrating all COPE skills as needed throughout the day. 

Similar to the rationale for practice of CDI and PDI skills, the use of TDI practice is expected to increase the likelihood that the SAFE and COPE skills will become rote and internalized for both carer and child. These skills will then become integrated into the family routine. Upon completion of the TDI phase of treatment, the family transitions into the PDI phase while continuing to incorporate the CDI and TDI skills learned.

#### 4.2.2. From the TDI Phase to the PDI Phase

Integrating TDI skills into the PDI phase begins by overlaying the previously learned trauma psychoeducation into the PDI teach. PCIT uses a positive discipline program which incorporates a structured time-out procedure [3]. Despite many scientific studies on the effectiveness of time-out, including with children who have experienced trauma [42,46,126], controversy and misinformation about this discipline strategy persist [127,128]. Consequently, when the idea of time-out is shared with carers, they may express concerns because of their child’s history. To avert this potential obstacle, in TDI, the time-out chair is referred to as the “calm-down chair,” as an acceptable tailoring of the PDI vernacular [129]. 

During the PDI teach, carers are reminded of the gains they have made in developing a more secure and warm relationship through their use of CDI skills, creating one of the strongest protective factors for children who have experienced trauma [108]. Additionally, the PDI teach reinforces the insights carers have developed throughout TDI to differentiate trauma activators from upset, as well as underscores their significant gains made thus far in PCIT. Specifically, carers are now well-versed in knowing how to respond to their child’s trauma distress and their own distress through their use of TDI skills. With the TDI adaptation, carers are provided a decision framework to uniquely address trauma reactions with or without an accompanying misbehavior. Additionally, the COPE skills are encouraged as mechanisms for the *carer* to calm *themselves* during emotionally intense portions of the PDI sequence (e.g., when the child yells or cries from the calm-down chair, the carer may be coached to practice color breathing). As PDI progresses, CDI skills continue to be integrated per the PCIT protocol, and the TDI skills are used as the opportunities arise. For example, therapists actively coach carers to first differentiate between child upset versus trauma activation as the situation presents and then guide carers in using TDI skills, such as color breathing and positive actions (e.g., distraction with an adult color sheet), to help themselves remain calm. Similar to TDI, SUDS ratings are used throughout PDI to gauge the carer’s experienced level of distress and to effectively provide responsive coaching. Likewise, homework continues to include CDI and TDI skills as families advance through PDI. No other changes to the PCIT discipline procedure are made. 

As PDI reinforces consistency, predictability, and follow-through with exact words to use, carers are expected to find the PDI procedure familiar as they have practiced such actions in TDI. The experience of a consistent carer response and scripting reinforced by coaching in the TDI phase will likely aid carers in the uptake and application of the highly regimented PDI phase, perhaps increasing acceptability of the PDI phase and facilitating attainment of graduation criteria. A reassessment of standardized measures and standardized carer-child interactions is completed at the conclusion of PCIT, with all the positive changes shared and celebrated with the family. Carers are strongly encouraged to continue using all they have learned in PCIT to maintain the gains the entire family has made over the course of treatment. 

## 5. Conclusions and Next Steps

In conclusion, the TDI module is consistent with the theory and the implementation of PCIT, incorporating the key elements of PCIT into this adaptation. With TDI, concerns from families, therapists, agencies, funders, and other stakeholders about PCIT’s ability to directly attend to trauma issues will be addressed. Importantly, with TDI, PCIT clinicians can consistently approach work with children who have experienced trauma and their carers who may also be impacted by trauma. This consistency is critical to the scientific evaluation and replication of PCIT for use with children with a trauma history. With the consistency in place, next steps in research are possible. A comparison of (1) standard PCIT to (2) PCIT with TDI will be a logical first step. A similar evaluation of standard PCIT to an adaptation with Mexican American families [104] found both arms to be equally effective in addressing presenting problems. This methodology allowed therapists to assess which PCIT application would be the best fit for families. If both standard PCIT and PCIT with TDI are shown to be equally effective for a population with trauma history, the best fit for all involved can be determined. 

It is recommended that outcome measures include assessment of (a) presenting problems, (b) trauma symptoms in both child and carers, (c) carer stress, and (d) treatment satisfaction of both carers and therapists. If TDI, as predicted, has an impact on the child’s behaviors and trauma symptoms and also reduce the carer’s stress and trauma symptoms, the field of childhood trauma and PCIT as an evidenced-based treatment will both be advanced. Attrition in child mental health services is high [130,131] with children having a history of maltreatment leaving treatment earlier and without successful changes than children without maltreatment [132]. Therefore, future studies of TDI should assess attrition. Relatedly, another area to examine in the evaluation of TDI is uptake of skills. As TDI teaches carers a consistent and predictable script to address trauma activators, it is predicted that carers’ learning of the PDI script, thus the time to PDI skills criteria, will be shorter than without TDI. Moreover, through clarifying upset versus trauma activators and developing coping skills for both child and carer, the intervening phase of TDI also may increase the acceptability of PDI by carers and clinicians alike for children with trauma. In current times, the need for mental health services for children has increased [84,85,86]. TDI may prove one more essential intervention for mental health professionals working with families and will help to meet the goals put forth by the U.S. Surgeon General’s Advisory [88] addressing the mental health crisis in youth. 

Finally, if carers find significant gains and report satisfaction with treatment for their children who have experienced trauma, they may be more amenable to a trauma-focused individual treatment to directly address their own trauma, given the large proportion of adult health issues related to ACEs [133]. When their trauma is addressed, carers report decreases in stress as well as in reported behavior problems in their children [134,135]. It is hypothesized that gains made from participation in PCIT plus TDI would be augmented in adult trauma treatment, leading to healthier family relationships. In the end, with the addition of TDI to the PCIT field, the ability to address trauma with PCIT will go from an “n of a million ones” to an “n of one million”.

## Figures and Tables

**Table 1:** PCIT as usual component comparison to TDI.

PCIT as Usual	TDI
Carer/Child Together	Yes
Coding	Yes
Coaching	Yes
Assessment-Driven	Yes Carer and child trauma assessments added
Skills Criteria	Yes (retained for CDI and PDI) Not required for TDI (time-limited)
Theory-Driven (attachment, developmental, behavioral)	Yes
CDI/PRIDE	Yes
PDI/Time Out and Back-Up	Yes
Homework	Yes
Phasal Sequence: (1) CDI (2) PDI	Phasal Sequence: (1) CDI (2) TDI * (3) PDI

* Trauma-Directed Interaction (trauma psychoeducation, COPE skills, and SAFE skills).

**Table 2:** Components introduced in TDI teach.

***Psychoeducation***
Define trauma
Discuss impact of trauma on brain development, behavior, relationships, and caregiving
Identify trauma activators
Differentiate upset vs. trauma activation
Build resilience
Introduce carer SUDS
***SAFE Skills (Carer Response)***
**S**tate the feeling
**A**ddress safety
**F**ind a COPE skill
**E**ngage in PRIDE skills
***COPE Skills (Child Response)***
**C**olor breathing
**O**pen to feelings
**P**ositive action
**E**xpress yourself

**Table 3:** TDI session progression.

Component	PCIT + TDI
Intake and Pretreatment DPICS	1–2 Sessions
CDI Teach	1 Session
CDI Coach	Sessions as needed to skills criteria
TDI Teach	1 Session Psychoeducation SAFE Skills COPE Skills
TDI Coach	3 Sessions TDI Coach 1: Color Breathing TDI Coach 2: Open to Feelings TDI Coach 3: Positive Action and Express Yourself
PDI Teach	1 Session
PDI Coach	Sessions as needed to graduation criteria
Graduation & Posttreatment DPICS	1–2 Sessions

**Table 4:** Example TDI treatment session timeline.

*One Carer in Treatment*	
*Time Allotted*	*Content*
	***Check-in***
3–5 min	Discuss issues of personal concern and take initial SUDS rating
5 min	Review TDI homework sheet: CDI with color breathing
3–5 min	Engage child and carer in brief color breathing practice
10 min	Introduce child and carer to the O in COPE for open to feelings
	***Coding and Coaching***
5 min	Code carer 1 in CDI play with child
<1 min	Complete In Session CDI Skills Progress Record; give carer CDI feedback and set coaching goals within TDI framework
15–20 min	Coach carer 1 in CDI and TDI with child
	***Check-out***
5 min	Share DPICS and ECBI data and review progress made within session
5 min	Take final SUDS rating; set goals/assign homework (CDI with color breathing and open to feelings)
50–60 min	Total approximate time for session
